# Preparation and Characterisation of Highly Loaded Fluorescent Chitosan Nanoparticles

**DOI:** 10.5402/2011/246162

**Published:** 2011-11-17

**Authors:** Haliza Katas, Chan Mui Wen

**Affiliations:** Faculty of Pharmacy, Universiti Kebangsaan Malaysia, Kuala Lumpur Campus, Jalan Raja Muda Abdul Aziz, 50300 Kuala Lumpur, Malaysia

## Abstract

Chitosan (CS) nanoparticles have been developed as a versatile drug delivery system to transport drugs, genes, proteins, and peptides into target sites. Demands on fluorescent nanoparticles have increased recently due to various applications in medical and stem-cell-based researches. In this study, fluorescent CS nanoparticles were prepared by a mild method, namely, complex coacervation. Entrapment efficiency of sulforhodamine (SR101) loaded into CS nanoparticles was investigated to evaluate their capacity in incorporating fluorescent molecule. Particle size of produced fluorescent nanoparticles was in the range of 600–700 nm, and their particle size was highly dependent on the CS molecular weight as well as concentration. A high entrapment efficiency of SR101 into CS nanoparticles could also be obtained when it was dissolved in methanol. In conclusion, highly loaded fluorescent CS nanoparticles could be easily prepared using complex coacervation method and therefore can be applied in various medical researches.

## 1. Introduction

In the last decade a strong emphasis has been given to the research and applications in the area of nanoscience and nanotechnology to bring advancement in the diagnosis and treatment of diseases. One of the focuses of nanotechnology is formulating therapeutic agents in biocompatible, polymeric, and submicron sized nanocomposites such as nanoparticles, nanocapsules, micellar systems, and conjugates [1]. 

Among polymeric nanoparticles available, chitosan nanoparticles have received a considerable amount of attention as a potential delivery system for drugs, genes, proteins, and peptides by different routes of administration. Chitosan (CS) is a polysaccharide that derived from deacetylation of chitin [2] and is a basic and positively charged hydrophilic polymer. The presence of amino group makes the CS easier to interact with various biomolecules such as protein, DNA, enzyme, and antibody. Besides, CS nanoparticles can also be used as a suitable matrix for attaching biomolecules and fluorescent molecules simultaneously [3]. 

CS nanoparticles can be prepared through several methods such as ionic gelation, complex coacervation, emulsion cross-linking, and spray drying [4, 5]. Among these methods, ionic gelation and complex coacervation are mild processes occurring in a pure aqueous environment [6], and the two methods are very similar except that ionic gelation involves the gelation of chitosan using an electrolyte such as tripolyphosphate (TPP), whereas the complex coacervation employs an oppositely charged ionic polymer [7] such as dextran sulfate (DS) [6]. 

The use of DS in the preparation of chitosan nanoparticles is gaining popularity as it is a biodegradable, biocompatible, and water-soluble polyanion. Besides, enhanced stability even in low pH [8] and increased mechanical strength are among the advantages of using DS as opposed to TPP which also highly influence the use of CS nanoparticles as a drug delivery system [4]. The combination of CS and DS as matrix materials to incorporate Rhodamine 6G has been reported, but these nanoparticles were negatively charged since nanosized particles could only be obtained when the charge ratio of sulfate (N) from DS and amino and imine groups (P) from Rhodamine 6G was 2.24 or greater [6]. Nanoparticles with positive value of surface charge are favorable in transporting any molecules into cells because, besides high loading capacity, a higher cellular uptake could be achieved compared with negatively charged nanoparticles [9] as opposite charges between nanoparticles and cell membrane would facilitate a better interaction and subsequently cellular internalization [10]. 

In this research, attempts have been made to produce positively charged fluorescent CS nanoparticles in which sulforhodamine 101 (SR 101), a water-soluble red fluorescent dye, was loaded into the nanoparticles. The need for high-capacity and easy-to-prepare fluorescent nanoparticles is paramount due to its various applications in medical and drug delivery research, for example, investigating neuronal morphology, monitoring regulated exocytosis and endocytosis [11–15] as well as in stem cells therapy, and acting as a labeling agent to allow visualisation of implanted cells [16]. 

The main objectives of this research therefore were to prepare and determine the entrapment efficiency of SR-101-loaded into CS nanoparticles, as well as to determine certain formulation factors that could affect their physical characteristics which include particle size, size distribution, and zeta potential.

## 2. Methodology

### 2.1. Materials

High-molecular-weight chitosan powder (HMW, molecular weight >310 kDa and degree of deacetylation >85%), low-molecular-weight chitosan powder (LMW, molecular weight of 50–190 kDa and degree of deacetylation 75%–85%), acetic acid, and sulforhodamine 101 were purchased from Sigma-Aldrich (USA). Dextran sulfate was obtained from Fisher Scientific (Canada), while methanol and acetone were obtained from R & M Marketing (Essex, UK). All solvents used were of analytical grade.

### 2.2. Methods

#### 2.2.1. Preparation of CS-DS Nanoparticles

CS-DS nanoparticles were prepared by the complex coacervation of CS and DS. High-molecular-weight (HMW) and Low-molecular-weight (LMW) CS were used to prepare 0.2, 0.1, and 0.05% w/v CS solution by dissolving required amount of CS powder in 2% v/v acetic acid solution. DS powder was dissolved in distilled water to prepare 0.2, 0.15, 0.1, and 0.05% w/v DS solution. After that, each 1 mL of DS solutions was added to each 4 mL CS solutions under magnetic stirring by using WiseStir digital multipoint magnetic stirrer, MS-MP8 (DAIHAN Scientific, Korea) at 250 rpm for 15 minutes. All samples were made in triplicate.

#### 2.2.2. Preparation of SR-101-Loaded CS-DS Nanoparticles

SR 101 loading into CS-DS nanoparticles was performed by incorporation method. A SR101 solution of concentration 0.9 mg/mL was first prepared by dissolving SR101 either in methanol or directly in 0.1 or 0.05% w/v DS solution. A volume of 1 mL 0.1 and 0.05% w/v DS solutions containing SR 101 was then added to the 0.1 and 0.05% CS solution, respectively, under magnetic stirring as above. For the SR 101 dissolved in methanol, the solution was mixed with DS solution prior to adding to CS solution. All samples were made in triplicate.

#### 2.2.3. Nanoparticles Characterisation

Particle size, zeta potential (surface charge), and polydispersity (PDI) were measured using the Zetasizer Nano ZS (Malvern Instruments, UK). The particle size measurement was performed using a quartz cell. The particle size analysis of each sample was performed at 25°C with a detection angle of 90° with three repeated measurements. For zeta potential measurements, samples were measured under 25°C. All following Z-average was reported as mean particle sizes. The particle size distribution was reported as a polydispersity index (PDI). 

#### 2.2.4. Entrapment Efficiency of SR 101

The amount of SR-101-loaded in the nanoparticles was calculated by the difference between the total amount SR 101 added to the nanoparticles formation medium and the amount of free SR 101 remaining in the aqueous supernatant. The latter was determined following the separation of SR 101 loaded nanoparticles from the aqueous medium by centrifugation twice (Optima L-100 XP Ultracentrifuge, Beckman-Coulter, USA, rotor NV 70.1 Ti, Beckman-Coulter, USA) at 15000 rpm and 4°C for 15 minutes. The SR 101 in supernatants was measured by UV-Vis spectrophotometer (double-beam ultraviolet visible spectrophotometer model UV-1601, Shimadzu, Japan) at the wavelengths of 492 and 536 nm for the nanoparticles in which SR 101 dissolved in methanol and distilled water, respectively. The SR 101 entrapment efficiency was calculated from the following equation:

(1) $$\begin{array}{cc} & \frac{\text{amount}\;\text{of}\;\text{SR}\;101\;\text{added}}{\text{amount}\;\text{of}\;\text{SR}\;101\;\text{added}}\\ & \;\;-\frac{\text{amount}\;\text{of}\;\text{free}\;\text{SR}\;101\;\text{in}\;\text{supernatant}}{\text{amount}\;\text{of}\;\text{SR}\;101\;\text{added}}\times 100. \end{array}$$

#### 2.2.5. Statistical Analysis

Data were summarized as the means ± standard deviation (SD). Data were analysed with independent *t*-test or one-way ANOVA by using SPSS 17.0. For independent *t*-test and one-way ANOVA, *P* values of <0.05 were considered as statistically significant which were different between the groups tested. 

## 3. Results and Discussions

The main aim of this study was to load-water soluble SR 101 into CS nanoparticles via complex coacervation method. The opposite charges of CS and DS were responsible for the formation of nanoparticles. DS was used in the study as the oppositely charged polymer because of its higher anionic charge densities (DS has 2.3 negative charges per monomer whereas CS contains 0.85 protonable amino groups per monomer) which therefore could provide sufficient anionic charge to cross-link CS by electrostatic force [17]. Furthermore, the formation of nanoparticles was strongly influenced by the molecular weight and concentration of CS and DS. 

### 3.1. Effect of DS and CS Concentrations on Unloaded CS Nanoparticles

Table 1 represents the data on the effects of different concentrations of DS on physical characteristics of nanoparticles made from 0.1% w/v LMW CS. DS concentration of 0.2% w/v produced nanoparticles with the largest particle size and least homogenous. It was also observed that this formulation produced the least stable nanoparticle system as phase separation occurred after a while. In contrast to that, nanoparticles with the size less than 400 nm were obtained when 0.15, 0.1, and 0.05% w/v DS were mixed with 0.1% w/v LMW CS. On the other hand, zeta potential of CS nanoparticles increased with the reduction of DS concentrations (one-way ANOVA, Tukey's post hoc analysis, *P* < 0.05, Table 1). This, therefore, suggested that CS nanoparticles were more stable when DS concentration is in the range of 0.15–0.05% w/v. Improvement in physical stability of CS nanoparticles with the decrease in concentration of DS was thought to be due to their high positive zeta potential which makes these particles tend to repel with each other and there will be no tendency for them to aggregate. 

Different from LMW CS, particle size of most formulations that were produced from HMW CS was beyond nanosize range. Particle size of all formulations was more than 1 *μ*m when 0.1% w/v of CS was used (Table 2). It was also observed that double layers were formed when 0.05% w/v DS was used which suggests that this nanoparticulate system was physically unstable. Similar results were also obtained for 0.2% w/v CS solution which produced large nanoparticles, between 4 and 7 microns (data was not shown). The same trend seen previously with the LMW CS for the zeta potential of these nanoparticles when DS concentration was reduced (one-way ANOVA, Tukey's post hoc, *P* < 0.05). 

Furthermore, a smaller particle size could only be obtained when 0.05% w/v CS was used, and the smallest particle size was obtained when DS concentration was 0.15% w/v. Although it produced the smallest particle size, the nanoparticles were negatively charged. The negative value of this nanoparticulate system was thought to be due to the high concentration of DS compared to others as DS carries negative charges. DS concentrations less than 0.15% w/v produced nanoparticles with positive surface charge.

### 3.2. Incorporation of SR 101 into CS-DS Nanoparticles

In this experiment, two formulations were used: (1) 0.1% w/v of DS with 0.1% w/v LMW CS and (2) 0.05% w/v DS with 0.05% HMW CS. They were chosen based on the formulations that produced particle size in the desired size range for both CS molecular weights and with the positive zeta potential. The value of zeta potential preferably is less than 60 mV. A very high zeta potential value was avoided to reduce risk of cytotoxicity. SR-101-loaded CS nanoparticles were obtained spontaneously upon the mixing of the DS solution with the CS solution under magnetic stirring. The incorporation of SR 101 into the HMW CS nanoparticles resulted in a decrease in particle size when compared to the empty CS nanoparticles (Table 3). In contrast to that, increased particle size was observed when SR101 was incorporated into the LMW CS nanoparticles. Despite increment in size, particle size of LMW CS was smaller than the HMW CS nanoparticles. This was thought to be due to the decreased viscosity of the LMW chitosan which resulted in more efficient interaction between negatively charged DS and cationic Cs. However, incorporation of SR101 into these nanoparticles could not be achieved as their entrapment efficiency was less than 10% for both formulations. This was expected to be due to the instability of SR 101 in water which, therefore, could not be efficiently loaded into the nanoparticles. 

Even though SR 101 is soluble in water, it is not stable in water (measured pH of about 6) as its stability decreases in acidic condition and most stable at pH 8.5 [18]. The above nanoparticles, therefore, were reformulated by first dissolving SR 101 either in methanol or acetone. It was observed that SR 101 was fully dissolved in methanol and formed deeply coloured solution but partially dissolved in acetone. As SR 101 was fully dissolved in methanol and the measured pH of methanol was 8.6, SR 101 was, therefore, dissolved in methanol prior to preparing nanoparticles. The solution was then mixed with DS solution before adding the mixture into CS solution under magnetic stirring in which the loaded nanoparticles were spontaneously formed. 

As shown in Table 3, the incorporation of SR 101 in methanol into the CS-DS nanoparticles resulted in increase in particle size of the nanoparticles compared with the empty nanoparticles. This indicated that SR 101 was successfully incorporated into the CS nanoparticles. Besides, zeta potential for both formulations was lower than the previous formulations when the SR 101 was directly dissolved in DS solution. On the other hand, the results obtained for entrapment efficiency also suggested that by dissolving SR 101 in the basic methanol, increased amount of SR 101 could be loaded into nanoparticles. The entrapment efficiency of the two formulations was approximately 96%, indicating that almost all SR 101 added was incorporated into the CS nanoparticles. Ionic interaction between the sulfate groups of DS and amino as well as imine groups of SR 101 was expected to be the determinant for the SR 101 entrapment efficiency [6].

## 4. Conclusions

This study demonstrated that positively charged fluorescent CS nanoparticles could be obtained by a mild method like complex coacervation, and a high entrapment efficiency of fluorescent molecules in CS nanoparticles could be achieved by dissolving SR 101 in a basic medium, for example, methanol. Additionally, a smaller particle size of fluorescent CS nanoparticles could be obtained by using LMW CS and adjusting CS concentration. It is also suggested that CS-DS nanoparticle is a versatile nanoparticulate system which could be used in various medical applications.

## Figures and Tables

**Table 1:** Mean particle size, PDI, and zeta potential of 0.1% w/v LMW CS nanoparticles at different concentrations of DS, *n* = 3.

Concentration of DS (% w/v)	Mean particle size (nm) ±SD	Mean (PDI) ±SD	Mean particle zeta potential (mV) ±SD
0.2	864.1 ± 437.98	0.7 ± 0.13	+36.8 ± 0.29
0.15	396.1 ± 17.00	0.4 ± 0.04	+49.3 ± 1.39
0.1	364.0 ± 15.21	0.4 ± 0.02	+56.6 ± 4.09
0.05	316.4 ± 54.86	0.4 ± 0.07	+62.4 ± 0.42

**Table 2:** Mean particle size, PDI, and zeta potential of 0.05 and 0.1% w/v HMW CS nanoparticles at different concentrations of DS, *n* = 3.

	0.05% w/v CS			0.1% w/v CS		
[DS] (% w/v)	Mean particle size (nm) ±SD	Mean PDI ±SD	Mean zeta potential (mV) ±SD	Mean particle size (nm) ±SD	Mean PDI ±SD	Mean zeta potential (mV) ±SD
0.15	373.1 ± 23.33	0.3 ± 0.05	−32.5 ± 1.23	1082.1 ± 245.67	0.90 ± 0.08	+72.5 ± 4.27
0.1	1141.3 ± 987.10	0.4 ± 0.13	+36.4 ± 2.39	1532.7 ± 284.65	0.98 ± 0.01	+84.8 ± 6.69
0.05	603.4 ± 27.90	0.8 ± 0.12	+78.1 ± 1.46	1439.7 ± 129.74	1.00 ± 0	+89.2 ± 2.98

**Table 3:** Effect of solvent used to dissolve SR101 on mean particle size, PDI, and zeta potential of SR-101-loaded CS nanoparticles, *n* = 3.

	Distilled water			Methanol		
	Mean particle size (nm) ±SD	Mean (PDI) ±SD	Mean zeta potential (mV) ±SD	Mean particle size (nm) ±SD	Mean (PDI) ±SD	Mean zeta potential (mV) ±SD
0.1% DS & LMW CS	394.07 ± 23.29	0.38 ± 0.02	+54.30 ± 1.18	620.03 ± 185.16	0.51 ± 0.14	+33.68 ± 16.46
0.05% DS & HMW CS	499.17 ± 21.22	0.67 ± 0.12	+69.50 ± 1.70	687.53 ± 222.36	0.59 ± 0.18	+44.78 ± 16.31
